# Standardized and Experimental Tools to Assess Spatial Cognition in Visually Impaired Children: A Mini-Review

**DOI:** 10.3389/fnins.2020.562589

**Published:** 2020-09-16

**Authors:** Giorgia Aprile, Giulia Cappagli, Federica Morelli, Monica Gori, Sabrina Signorini

**Affiliations:** ^1^Centre of Child Neurophthalmology, IRCCS Mondino Foundation, Pavia, Italy; ^2^Unit for Visually Impaired People, Istituto Italiano di Tecnologia, Genova, Italy

**Keywords:** spatial cognition, visual impaired children, clinical assessment, review, rehabilitation

## Abstract

The acquisition of spatial cognition is essential for both everyday functioning (e.g., navigation) and more specific goals (e.g., mathematics), therefore being able to assess and monitor spatial cognition from the first years of life would be essential to predict developmental outcomes and timely intervene whenever spatial development is compromised. Several shreds of evidence have indicated that spatial development can be compromised in the case of development with atypical sensory experience such as blindness. Despite the massive importance of spatial abilities for the development of psychomotor competencies across childhood, only a few standardized and experimental methods have been developed to assess them in visually impaired children. In this review, we will give a short overview of current formal (standardized) and informal (experimental) methods to assess spatial cognition in visually impaired children, demonstrating that very few validated tools have been proposed to date. The main contribution of this current work is to highlight the need of *ad hoc* studies to create and validate clinical measures to assess spatial cognition in visually impaired individuals and address potential future developments in this area of research.

## Introduction

Several shreds of evidence have shown that the acquisition of spatial capabilities is fundamental for psychomotor, cognitive, and social development (Newcombe and Huttenlocher, 2000; Vasilyeva and Lourenco, 2012). For instance, it has been suggested that spatial cognition not only is fundamental to navigate and orient in the environment, even with the use of symbolical means such as models or maps (Montello and Raubal, 2012), but also it is linked to perspective taking and problem-solving (Newcombe and Learmonth, 2009). Overall, spatial cognition refers to the knowledge and internal or cognitive representation of the structure, entities, and relations of space (Hart and Moore, 2017). In this sense, spatial cognition can be considered as a component of the broader concept of intelligence and constitutes a relevant element that defines the structure of human intellect, involved both in everyday life skills and in the acquisition of learning abilities (Newcombe et al., 2013). The neuropsychological examination of general intelligence in children and adults typically takes into consideration spatial subtests. For instance, the most recent version of the Wechsler Intelligence Scale for Children includes a subtest (Visual Spatial Index – VSI) that directly assesses visuo-spatial processing. Also, many tests have been developed to specifically assess different aspects of spatial cognition in sighted children, such as visuo-spatial perception, visuo-spatial memory, construct abilities and orientation skills. Such findings suggest that a crucial factor in fostering psychomotor and cognitive development is spatial cognition, which supports the acquisition of fundamental abilities such as reasoning. This argues in favor of the importance of an overall assessment of such abilities to better define the cognitive profile of the child, whether he/she is sighted or visually impaired. In literature, the evaluation of spatial cognition in visually impaired people has led to contrasting results: some work supports the hypothesis of visually impaired people being able to acquire similar spatial competencies to their sighted peers, while several other works have indicated that spatial cognition can be compromised in visually impaired people (Thinus-Blanc and Gaunet, 1997; Pasqualotto and Proulx, 2012; Schinazi et al., 2016; Giudice, 2018). In this sense, the assessment of spatial abilities in this population would be necessary to personalize rehabilitation interventions based on patients’ strengths and limitations. Indeed, children with visual impairment may manifest delays in locomotion development (Hallemans et al., 2011) and difficulties in understanding the topographical position of sound sources in space (Fazzi et al., 2011; Cappagli and Gori, 2016; Vercillo et al., 2016) as well as in proprioceptive localization (Cappagli et al., 2017a). Nonetheless, very few standardized tools exist to assess spatial abilities in children with blindness or low vision. The reason could be that most of the conventional spatial tests for typical children are visual and cannot be easily adapted to other modalities (such as hearing or touch) for technical reasons. On the contrary, many research paradigms have been developed to assess spatial cognition in children with none or partial vision, but none of them has been formally validated, except for a single study, to our knowledge, that developed and validated an experimental battery of spatial tests, providing the first gold standard for assessing spatial cognition deficits in visually impaired children (Finocchietti et al., 2019). This study evaluated the reliability of a test battery comprising six spatial tests assessing different spatial competencies (e.g., the ability to estimate the topographical representation of single or multiple sound sources) on a group of thirty aged 6–17 visually impaired children and showed good-to-excellent reliability for all six tests. From a practical point of view, the lack of standardized methods to assess spatial cognition in children with visual impairment results in a lack of outcome measures for rehabilitation interventions, limiting the objective evaluation of children’s advancements, as highlighted in a recent review on the topic (Elsman et al., 2019).

## Standardized Tools to Assess Spatial Cognition in the Visually Impaired Child

A range of psychometric tests has been developed for blind or partially sighted individuals. Some of them are based on tests for sighted people, while others have been specifically designed for visually impaired people. Tests developed for adults include, for example, the Cognitive Test for the Blind (Nelson et al., 2002) and the Haptic Intelligence Scale for Adult Blind (Shurrager and Shurrager, 1964). Since this mini-review is intended to summarize only tools designed for visually impaired children, a description of the adult tests goes beyond our purpose.

Regarding the standardized tools to assess spatial cognition in visually impaired children, we highlight some limitations: first of all, the majority of them have been developed as a general intelligence test, with spatial cognition representing only a limited part. Other limitations are the absence of validation measures and the lack of reported evidence about their acceptance rate (Atkins et al., 2012). Moreover, developmental tests to measure general intelligence and/or specific aspects of intelligence (i.e., spatial cognition) in visually impaired children are mostly for children in scholar age (from 6 years old) and lack of standardized norms or have norms that refer to sighted peers (Bauman and Kropf, 1979). Moreover, most of them are based almost exclusively on the assessment of haptic spatial processing, and are used to assess children with different degree of visual impairment: in this sense a low vision child could be disadvantaged, compared to a blind peer, when faced with a test requiring only haptic abilities, not being put in a position to use his/her residual vision.

Below we report a list of the most used standardized tests specifically targeted to measure spatial capabilities or more widely related to general intelligence in visually impaired children (in Table 1 we summarized the main studies reported below).

**TABLE 1:** List of standardized tests to assess spatial cognition in visually impaired children with their main related studies.

Test	Related studies	Visually impaired sample	Functional vision	Age	Comorbidities	Sighted controls (sample size)	Type of analysis	Main result	Limits
Reynell-Zinkin Scale *	Reynell (1978)	109	Blind and partially sighted	0–60 months	17 mild cerebral palsy 8 hearing impairment	Yes (NA)	Comparative quantitative study (visually impaired VS sighted)	A developmental pattern for visually impaired children is outlined with respect to sighted children	- Reliability and validity measures were not provided - Not specifically designed to assess spatial abilities
	Dote-Kwan (1995)	18	Blind	20–36 months	No	No	Pearson product-moment correlation	Strong correlation with Maxfield-Buchholz score	- Small sample size - Limited age range
	Vervloed et al. (2000)	82	Low vision	0–48 months	No	No	Study of item distribution, scale reproducibility, internal consistency, standard errors of measurement, and associations with personal and environmental factors.	New developmental age levels were provided	- Internal consistency of the subscales is not sufficient at all ages for all the subscales
Haptic Test Battery	Ballesteros et al. (2005)	59	Blind and partially sighted	3–16 years	No	Yes (60)	Alpha coefficient for reliability; factor analysis for validity	Good internal consistency, construct validity (six-factor structure) and age sensitivity	- Not specifically designed to assess spatial abilities - Assessment of only haptic spatial processing - No differences between blind and low vision children in test administration nor specific adaptations
Haptic 2-D	Mazella et al. (2016)	69	Blind and low vision	5–25 years	26 out of 69 with cognitive/sensory or neurological comorbidities	Yes (69)	Test-retest reliability; Convergent validity; Discriminant validity	Good sensitivity, reliability and validity. Better suited to assess tactual abilities at 5 to 18 years old	- Not specifically designed to assess spatial abilities - Assessment of only haptic spatial processing - No differences between blind and low vision children in test administration nor specific adaptations - Only one-hand exploratory movements
The Bayley Scales Of Infant And Toddler Development (3rd Edition) **	Bayley (2006)	1700	Typically developing children (standardization sample)	1–42 months	10% with mental, physical and behavioral difficulties	No	Split-half method and coefficient alpha for internal consistency reliability; Factor analysis	Good validity and reliability	- Not specifically designed to assess spatial abilities in visually impaired children - Potential floor inadequacy for lower performing and extremely young children (0–6 months)
	Visser et al. (2014)	19	11 out of 19 with low vision	22–90 months	All 19 children with different degree of motor impairment	No	Comparative quantitative study (standard test VS accomodated test)	No significant different between Low Motor/Vision accommodated version and the standard version. Accommodations showed to be beneficial for a subgroup of children	- No vision adaptation for blind children (i.e., tactual or sonorous) - Small sample size - No data to support the hypothesis of a better performance with the adapted test
Intelligence Test For Visually Impaired Children	Dekker (1993)	155	106 blind children, 49 visually impaired children	6–16 years	No	No	Alpha coefficients and odd-even split-half coefficient for reliability; Factor analysis	Good validity and reliability. Useful to compare braille-educated and print-educated visually impaired children	- Not specifically designed to assess spatial abilities - Assessment of only haptic spatial processing - Separated age-normed tables should be used for children with and without usable vision
The Hill Performance Test Of Selected Positional Concepts	Hill and Hill (1980)	273	Blind	6–10 years	64 with additional disabilities (no further information available)	No	Test-retest method for reliability; Spearman Rank-Order Correlation for validity	Sufficiently reliable and valid	No assessment of spatial concepts other than basic positional concepts

*We included the original study (Reynell, 1978), the first study (Dote-Kwan, 1995) that provided some quantitative data about the validity of the scale and the study (Vervloed et al., 2000) that provided new developmental age levels for the Reynell-Zinkin Scale. **We provided both the standardization study of the third edition (Bayley, 2006) and the pilot study (Visser et al., 2014) that tested specific adaptation for visual and motor disabilities.

### Reynell-Zinkin Scales (Reynell and Zinkin, 1975; Reynell, 1978)

This test was designed to assess the motor and mental development of blind or partially sighted children from birth to 5 years of age. The original study (Reynell and Zinkin, 1975) was based on 116 recordings of a heterogeneous population of visually impaired children (blind or partially sighted) with any other disabilities, whose both motor and mental development were assessed. Subsequently, the same authors (Reynell, 1978) used the Reynell-Zinkin Mental Development Scale to assess a more homogeneous population composed of 109 visually impaired children almost without associated disabilities (17 had mild cerebral palsy, 8 had hearing impairment). Nonetheless, no reliability and validity data were available until 1995 (Dote-Kwan, 1995). Vervloed et al. (2000), given the lack of psychometric data in the original version of the scale and the clinical impression that the age norms tended to overestimate the real developmental level of visually impaired children, constructed new age developmental levels. In this study, 82 children aged 0–48 months with low vision without any other disability were included. It was concluded that the Reynell-Zinkin Scales, to evaluate the rate of progress of visually impaired children, should be better administered between ages 1 and 3.5 years old. Also, the authors pointed out that: (a) these new age levels should not be used to compare the separate scales due to the lack of standardized scores; (b) with the new age levels, it was possible to compare the rate of development in similar visual categories. By now, the Reynell-Zinkin Mental Development Scale focuses on six developmental areas, each assessed through specific items: social adaptation, sensorimotor understanding, exploration of the environment, response to sound and verbal comprehension, expressive language, and communication. The purpose of these scales is to define, as early as possible, the abilities and difficulties of a visually impaired child to better tailor an appropriate intervention. The assessment of spatial abilities is better represented by the “exploration of environment” scale: the items proposed are intended to progressively assess the ability to explore surfaces and objects encountered during locomotion, orient in a room and explore it through directed locomotion, also using fixed objects (doors, pegs or other furniture) as reference points. Moreover, the subscale “response to sound” gives indication about children’ ability to orient to sonorous stimuli in space.

### Haptic Test Battery (Ballesteros et al., 2005)

This test has been developed to assess the perceptual and cognitive abilities of visually impaired children without any other known disabilities between 3 and 16 years old using active touch. First designed in 2002, it was subsequently refined (Ballesteros et al., 2005) to examine its reliability and construct validity on 119 schoolchildren (59 blind or visually impaired, 60 sighted). The original battery included twenty sub-tests. The first three sub-tests were adapted from the Luria-DNI neuropsychological battery (Manga and Ramos, 2017). The others explored perception of texture, 2-D raised-line shapes, and 3-D objects, as well as the perception of their spatial orientation, and memory for familiar and for novel objects, recognized in previous studies as major aspects of haptic perception and cognition (Lederman, 1983; Millar, 1986; Ballesteros et al., 1999). Test materials are composed by raised-line, raised-dot, raised-surface shapes and displays, and familiar and novel 3-D objects. At the end of the reliability and validity study, six factors were identified as being explored by the sub-tests proposed. The first one, spatial comprehension, includes seven sub-tests that aim to assess, respectively: (i) the ability of the child to compare different haptic dimensions (shape, size, texture), (ii) to recognize shapes with the same orientation, (iii) to individuate spatial location on a 2D surface, (iv) to classify objects as “symmetric” or “asymmetric” (three subtests), and (v) to recognize “non-sense” 3D objects after an interpolated task (this last sub-test is also included in the “Longer-term coding for new objects” factor). Regarding the statistical influence of age and visual condition, age resulted in being significant in all the subtests, and the visual condition showed to be significant in five sub-tests (in which blind performed better than the sighted group). The authors concluded that all these sub-tests investigate aspects of spatial perception and cognition, resulting in a valid assessment of haptic spatial processing and development. In our opinion, two subtests assessing, respectively, the ability to recognize and localize stimuli on a worksheet and the ability to scan a dot-display (both involved in pre-school ability to read braille) may be useful to evaluate children’ spatial abilities, giving the importance of such abilities in the development of good pre-school skills.

### Haptic-2D (Mazella et al., 2016)

This is a psychometric test originally designed for visually impaired subjects between 5 and 18 years of age. In the validation study, subjects with additional disabilities were included in the visually impaired group. According to the authors, it is the first test taking a developmental approach to tactual abilities using 2D raised materials only (dots, lines, shapes, patterns, and pictures printed on swell paper). The purpose of the battery is to evaluate tactile functioning in terms of raised-shape processing, sequential scanning and raised-line object identification, thus providing information about essential pre-school and everyday life abilities. The battery is composed of eleven tests divided into five categories: (a) scanning skills, (b) tactile discrimination skills, (c) spatial comprehension skills, (d) short-term memory, (e) picture comprehension. Some of the tests are taken or adapted from Haptic Test Battery (Ballesteros et al., 2005). The two tests included in the spatial comprehension dimension are, respectively, a spatial orientation and a spatial location test. In both tests, the participant is presented with a series of six items, preceded by a practice trial, with the aim of comparing the tactile item with a benchmark stimulus. The authors concluded that the haptic modality can be used in the context of psychometric evaluations for visually impaired or blind children and adolescents, providing a measure of the age-related efficiency of processing of raised materials. According to the authors, the proposed battery has good psychometric properties but shows two major limitations, one referred to the constraints imposed on the exploratory hand movements (most of the tests have to be performed with one hand only), the other referred to the possibility of using non-informative vision during the tests by subjects with low or normal vision. Subjects with additional disabilities were included in the original study group of 138 participants, showing significantly different scores respect to subjects with no additional disorders only in the tactile memory span test.

### The Bayley Scales of Infant and Toddler Development (Bayley, 1969)

In its third version, this is an instrument designed to measure the developmental functioning of infants and toddlers and to identify possible developmental delay (Albers and Grieve, 2006). The original Bayley Scales of Infant Development (BSID) was published in 1969 (Bayley, 1969), while the second edition (BSID-II) was presented in 1993 (Bayley, 1993). The Bayley-III is designed to be administered to children between 1 and 42 months of age and is composed of Cognitive, Language, Motor, Social-Emotional, and Adaptive Behavior scales. Spatial perception evaluation can be found in the Cognitive and the Motor scales, where visual and tactile exploration and perceptual-motor integration tasks are proposed (e.g., building simple structures, tracing an outline on paper, visual tracking, reaching). The Scale is designed to be used as part of the assessment for children with various disorders and/or disabilities. In 2014 (Visser et al., 2014), a pilot study was conducted based on an adapted version of the test for children with visual and/or motor impairment to increase the construct validity by decreasing the influence of the impairment on the test results. The adaptation was based on the Low Motor and Low Vision accommodated versions of the Dutch Second Edition of the Bayley Scales of Infant Development (Ruiter et al., 2011). The Low Vision adaptations were integrated with the Low Motor adaptations and consisted of slight adaptations in materials and instructions and in the removal of the time limits, allowing children to have more time to explore and identify materials. Children were administered once the Low Motor/Vision accommodated version and once the standard version of the Bayley-III, with an average time interval of 2 weeks between the two administrations. The authors concluded that some of the children with motor and/or visual impairment might benefit from the adaptations, which result in a smoother test administration and valid test results, even though there were no significant data to support the hypothesis that most of the children would score higher with the adapted test.

### Intelligence Test for Visually Impaired Children (Dekker, 1993)

This tool was developed to fill the gap between the need to measure intelligence in visually impaired children aged between 6 and 15 years old and the lack of instruments specifically designed for this population. It was designed to include 13 subtests assessing a broad spectrum of abilities, from verbal competencies to reasoning, memory skills, and spatial perception. Regarding the latter, four subtests were identified as underlying, respectively, the Orientation factor (“Map Question” and “Plan Questions”) and the Spatial Ability factor (“Block Design” and “Rectangle Puzzles”). Dekker (1993) administered this test to a Dutch-speaking population of children between 6 and 16 years old with “usable” and “no usable” vision. An analysis of the results depending on the grade of visual impairment and the visual education received (“print” or “braille”) was performed. Results indicated that low-vision performed better than blind children in spatial and orientation sub-tests. Moreover blind children referred to braille-education at the age of six obtained the lowest scores in the spatial subtests, indicating these two variables have to be taken into consideration when the test is administered.

### The Hill Performance Test of Selected Positional Concepts (Hill and Hill, 1980)

This test is based on the “Concepts Involved in Body Position and Space” test developed by the same author (Hill, 1971) to evaluate basic positional concepts in visually impaired children between 6 and 10 years of age. The seventy-two performance items are divided into four parts assessing the abilities to (a) identify positional relationships of body parts; (b) demonstrate positional concepts by moving various body parts to one another; (c) demonstrate positional concepts by moving the body in relation to objects; (d) form object-to-object relationships. The revised test was validated on a sample of 273 American visually impaired children with basic skills regarding motion, body parts knowledge, and receptive language, and was administered by orientation and mobility specialists. The authors did not find a significant difference in performance according to the school placement, nor to the ability to read braille. To our knowledge, no studies were published using this test.

## Research Paradigms to Assess Spatial Cognition in the Visually Impaired Child

In contrast with the very few standardized methods listed above, many experimental paradigms have been developed to investigate spatial cognition in visually impaired individuals, but most of them have been tested only on adults, leading to mixed results (Thinus-Blanc and Gaunet, 1997; Pasqualotto and Proulx, 2012; Voss, 2016; Setti et al., 2018). The criteria generally used by researchers to define experimental paradigms are very different among studies. A useful distinction can be made between locomotion tasks and tabletop tasks, respectively, requiring and not requiring individuals to move in the environment. Both intended to test the ability of participants to build a spatial representation of the setup (Klatzky et al., 1995). It is not the purpose of this work to review all the research paradigms proposed in the literature, but to provide examples of experimental tabletop and locomotion tests designed for visually impaired children that might be taken as a reference for future validation studies. Moreover, it would be useful to link novel validated experimental paradigms to standardized clinical tools in order to provide clinicians and researchers a comprehensive battery of methods to assess spatial cognition in the visually impaired child.

Among tabletop tests, different auditory and haptic spatial competencies have been investigated in visually impaired children. For instance, the ability to understand the spatial relation of three sounds differently positioned in space (Vercillo et al., 2016) and the ability to identify and/or reproduce the spatial position of auditory (Ashmead et al., 1998; Cappagli and Gori, 2016; Cappagli et al., 2017a) or haptic targets (Gaunet et al., 2007; Ittyerah et al., 2007), the ability to switch from egocentric to allocentric spatial frames of reference (Ochaita and Huertas, 1993). Moreover, some research has shown that technological tools such as programmable tactile displays can be used to administer tabletop spatial perception and memory tests to visually impaired children (Leo et al., 2017, 2018). Among locomotion tests, visually impaired children were tested on their ability to detect and avoid obstacles (Ashmead et al., 1989), identify and reach sonorous objects (Bigelow, 1986; Ihsen et al., 2010; Fazzi et al., 2011), make spatial inferences finding new routes between external landmarks (Landau et al., 1984) and navigate in large-scale environments showing evidence of spatial cognitive mapping (Morrongiello et al., 1995).

To our knowledge, only one study validated a battery of experimental spatial tests (BSP, Blind Spatial Perception), providing the first gold standard for assessing spatial cognition deficits in visually impaired children (Finocchietti et al., 2019). A group of thirty children with visual impairments aged 6–17 years old were tested on the BSP comprising the following six spatial tasks: auditory bisection (listen to three sounds and report whether the second sound was closer in space to the first or to the last one presented), auditory localization (listen to one sound and point toward its spatial location), auditory distance discrimination (listen to three sounds and report whether the first or the second presented is closer to their body), auditory reaching (listen to one sound and reach it in space), proprioceptive reaching (repeat a memorized arm movement toward a specific spatial position), and general mobility (walk straight for three meters and come back at their own pace). Test-retest reliability showed good-to-excellent reliability for all six tests, demonstrating that the BSP is a reliable tool to identify spatial impairments in visually impaired children.

## Current Research Gaps and Potential Future Developments

It is well known that visual experience alone and multisensory experience involving visual information is crucial in forming a mental representation of space (Eimer, 2004; Pasqualotto and Proulx, 2012). Research studies suggest that the absence of vision affects the development of specific spatial abilities in visually impaired children (Cappagli and Gori, 2016; Vercillo et al., 2016; Cappagli et al., 2017a) with possible negative consequences on other developmental domains such as social cognition (Hestenes and Carroll, 2000; Lewis et al., 2000). This evidence leads to the hypothesis that rehabilitation programs should include specific multisensory spatial training from an early age (Cappagli et al., 2017b, 2019). Standardized clinical and/or experimental tools to assess spatial cognition in the developing visually impaired child would provide the criteria to identify both spatial challenges and rate of progress and consequently to focus habilitation intervention strategies on areas requiring support. Being able to identify spatial impairments with standardized and validated methods early would increase the benefits of rehabilitation interventions. With this review, we highlighted that very few standardized tools to assess spatial cognition in visually impaired children have been developed. Moreover, the tools developed to date present some important limitations: for example, the poor specificity for spatial cognition (most of them being primarily developed as general intelligence tests), the preeminent use of haptic modality, and the general lack of differentiation between blind and low vision children, not allowing for an adequate exploitation of residual vision. Furthermore, almost none experimental methods have been validated until now. The lack of standard and validated behavioral measures to evaluate spatial abilities in the visually impaired has led to contrasting results in the literature about enhancements and impoverishments of spatial cognition in this population. In this sense, our research group is currently working to develop assessment tools for spatial cognition that can be standardized on a visually impaired pediatric population, also through proper adaptations of the most commonly used tests for sighted children. Moreover, we are also working to increase scientific knowledge about the developmental aspects of spatial cognition in visually impaired children [e.g., Martolini et al. investigates allocentric spatial development in low-vision children (Martolini et al., 2020)].

Overall, with this work we identified two main research gaps that might address future development in this research area:

1. the lack of formal (standardized) tests to evaluate spatial capabilities in visually impaired children and/or the lack of auditory or tactile adaptations of existing standardized tools to assess the same skills in sighted individuals;
2. the lack of informal (experimental) validated methods to determine spatial cognition in the visually impaired pediatric population, especially for what concerns paradigms in the auditory modality.

Potential future developments in this area of research may concern not only the creation of new standardized test for spatial evaluation as suggested in the previous sections, but also the validation and large-scale application of recently developed prosthetic devices to support spatial learning in blind individuals. Indeed, during the recent years, there has been an increasing interest in the development of technological aids to convey spatial information to the blind by means of haptic (e.g., accessible interactive tactile maps for geographical representations) (Ducasse et al., 2018) or auditory (e.g., radio beacons for navigation or sound sensory substitution for obstacle avoidance) displays (Chebat et al., 2017; Strumillo et al., 2017). Such affordable devices have been created to allow visually impaired users to build a mental representation of the surrounding environment, fostering their overall adaptation to real-life situations. Nonetheless, very few of such technological tools have been validated on visually impaired children (Gori et al., 2016), with negative consequences on rehabilitation outcomes. Since it has been shown that technological systems to support spatial learning blind individuals can even determine training-induced plastic changes, it would be promising to expand their use in the visually impaired community. For instance, the validation of technical aids to support the acquisition of echolocation skills would be of fundamental importance for visually impaired individuals, since it has been shown that echolocators can develop sighted-like performance in terms of spatial cognition (Teng et al., 2012; Vercillo et al., 2015). Similarly, it would be interesting to validate systems that support spatial learning in blind children from an early age, since it has been shown that they can foster multisensory development such as the ABBI (Audio Bracelet for Blind Interaction) device (Finocchietti et al., 2015; Ben Porquis et al., 2018).

## Author Contributions

GA, GC, FM, and SS conceived the manuscript. GA, GC, and FM wrote the article. SS and MG provided critical feedback and contributed to the final approval of the submitted version. All authors contributed to the article and approved the submitted version.

## Conflict of Interest

The authors declare that the research was conducted in the absence of any commercial or financial relationships that could be construed as a potential conflict of interest.
